# Closure of enterotomy after side-to-side ileocolic anastomosis with two barbed sutures in totally laparoscopic colectomy for right-sided colon cancer

**DOI:** 10.1007/s00595-020-02108-1

**Published:** 2020-08-11

**Authors:** Hiroki Hamamoto, Junji Okuda, Keisuke Izuhara, Masatsugu Ishii, Wataru Osumi, Shinsuke Masubuchi, Masashi Yamamoto, Keitaro Tanaka, Kazuhisa Uchiyama

**Affiliations:** Department of General and Gastroenterological Surgery, Osaka Medical College, 2-7 Daigaku-machi, Takatsuki, 569-8686 Japan

**Keywords:** Intracorporeal ileocolic anastomosis, Enterotomy closure, Barbed suture

## Abstract

Good short-term outcomes of intracorporeal ileocolic anastomosis (IIA) in totally laparoscopic colectomy for right-sided colon cancer (TLRC) have been shown in many reports, but no standardized technique for enterotomy closure after stapled side-to-side ileocolic anastomosis has so far been established. We retrospectively compared the short-term outcomes between 13 consecutive patients receiving either TLRC with IIA by conventional enterotomy closure (*n* = 6) or closure of the enterotomy using two barbed sutures (CEBAS) (*n* = 7) from July 2019 to April 2020. No anastomotic bleeding or leakage was observed in either group. Time to enterotomy closure was significantly shorter with the CEBAS method (16.5 ± 3.7 min) than with the conventional method (24.5 ± 4.7 min, *p* = 0.0059). The CEBAS method in TLRC with IIA was thus found to be technically feasible and it might reduce the stress associated with intracorporeal enterotomy closure.

**Electronic supplementary material:**

The online version of this article (10.1007/s00595-020-02108-1) contains supplementary material, which is available to authorized users.

## Introduction

A large amount of evidence has been accumulated in support of laparoscopic-assisted surgery for colon cancer, and laparoscopic-assisted colectomy is now widely performed. In most institutions, the standard procedure for laparoscopic-assisted colectomy for right-sided colon (LARC) is extracorporeal ileocolic anastomosis (EIA) after a high ligation of the central vessels and complete mesocolic excision. However, more recently, intracorporeal ileocolic anastomosis (IIA) in totally laparoscopic colectomy for right-sided colon cancer (TLRC) has gained interest, and several studies have compared the two techniques, LARC with EIA and TLRC with IIA. Milone et al. [1] reported that TLRC with IIA is associated with an earlier recovery of the postoperative bowel function than LARC with EIA, and Oostendorp et al. [2] reported that TLRC with IIA was associated with a decreased length of hospital stay. Despite these good short-term outcomes, IIA has not yet been widely adopted. This might be because of the complicated intraabdominal procedure, especially the closure of enterotomy after side-to-side ileocolic anastomosis. Our institution started TLRC with IIA in 2013 and has since experienced many cases. We herein describe our technique for enterotomy closure using two barbed sutures.

## Materials and methods

As in our previous report [3], enterotomy after side-to-side anastomosis by a 60-mm linear stapler was conventionally closed using the Albert–Lembert method; a continuous suture using barbed thread and several interrupted sutures using absorbable thread. From December 2019, we started to perform the Closure of Enterotomy using Two Barbed Sutures (CEBAS) with the Albert–Lembert method. This retrospective study compared the short-term outcomes from 13 consecutive patients who received TLRC with IIA by a conventional enterotomy closure (*n* = 6) or CEBAS (*n* = 7) from July 2019 to April 2020. All surgical procedures were performed by the same surgeon (H.H.) with the colorectal surgical team.

This study was reviewed and approved by the institutional review board (IRB) at Osaka Medical College (IRB acceptance number: 2853) and it was performed in accordance with the Declaration of Helsinki. The statistical analysis was performed using the JMP version 14 software program (SAS Institute, Cary, NC, USA). Student’s *t-*test, the Mann–Whitney *U* test, and the *χ*^2^ test were used to compare continuous and categorical variables, as appropriate, with two-sided values of *p* < 0.05 indicating significance.

### Surgical Technique

**Fig. 1:**
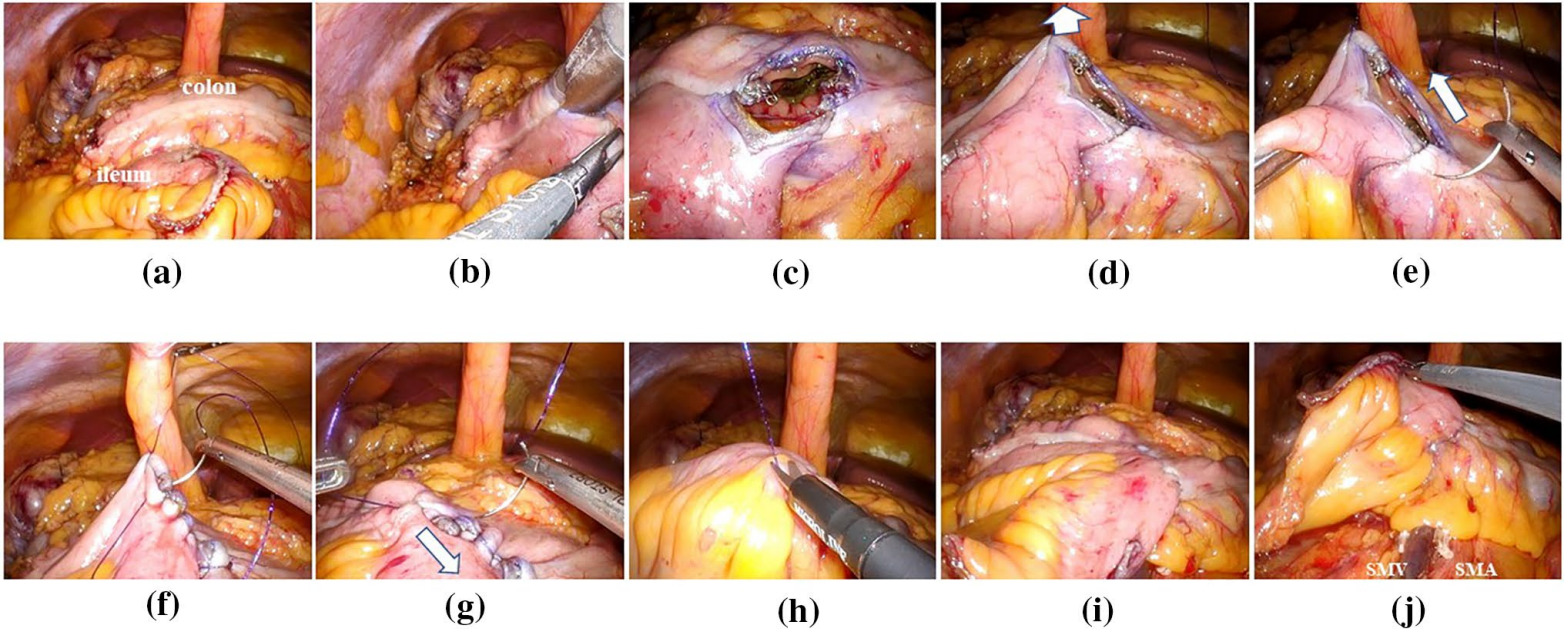
**a**–**c** Fashioning side-to-side ileocolic anastomosis with a linear stapler. **d** Pulling up in a cranial direction. **e**, **f** The first full-thickness inner layer closure is started from the proximal edge of the enterotomy. **g** The second seromuscular layer is started from the distal edge of the enterotomy. **h** After the last stitch of the seromuscular layer, the suture is cut. **i**, **j** The tightness of the anastomosis is ensured visually.

Five trocars were used in all procedures: a 12-mm trocar in the umbilical position, a 12-mm trocar in a suprapubic position, a 5-mm trocar in the right lower quadrant, a 5-mm trocar in the left lower quadrant, and a 5-mm trocar in an epigastric position. TLRC with IIA in our institution was performed as follows: (1) dissection of mesocolon from the retroperitoneum via Tolds fusion fascia (medial approach); (2) division of vessels according to tumor location (central vascular ligation); (3) hepatic flexure mobilization and dissection of lateral peritoneal attachments of the ascending colon (mobilization of the right-sided colon was complete); (4) division of the terminal ileum and transverse colon with linear staplers; (5) fashioning side-to-side ileocolic anastomosis with a 60-mm linear stapler in an isoperistaltic manner (Fig. 1a–c); (6) closure of the enterotomy with the Albert–Lembert method using a barbed suture and several absorbable sutures or two barbed sutures (the defect between the mesentery and mesocolon was not closed). We usually inserted the camera through a suprapubic trocar and operated with the left and right forceps through the trocars in the left and right lower quadrant when suturing; and (7) specimen extraction through a mini-laparotomy over the transumbilical port site (not the Pfannenstiel laparotomy) and finally the closure of skin incisions.

### Conventional method of enterotomy closure

After side-to-side anastomosis using a 60-mm linear stapler, a continuous suture using barbed thread was started from the proximal edge of the enterotomy to the distal edge. The second seromuscular layer was started from the distal edge to the proximal edge with several interrupted sutures using absorbable thread, so several ligations were needed when performing the conventional method.

#### CEBAS

The first barbed suture was used to pull up the distal edge of the enterotomy in a cranial direction (Fig. 1d). Full-thickness inner layer closure was started from the proximal edge of the enterotomy with another barbed suture toward the distal edge with a continuous technique (Fig. 1e, f). After the full-thickness layer was complete, the second seromuscular layer was started with the first barbed suture from the distal edge toward the proximal edge (Fig. 1g). After the last stitch of the seromuscular layer, the suture was simply cut without any ties (Fig. 1h). Finally, the tightness of the anastomosis was ensured visually (Fig. 1i, j) (Supplemental video 1).

## Results

### Clinical characteristics and short-term outcomes

The demographic and surgical data in the conventional and CEBAS groups showed no significant difference (Table 1). The results of short-term outcomes are also shown in Table 1. The first day of flatus, length of hospital stay, and rate of complications did not differ significantly between groups. Postoperative ileus occurred in one patient in each group, but no surgical site infection, postoperative bleeding or anastomotic leakage occurred in any group.

**Table 1:** Demographic and surgical data, and the short-term outcomes

	Conventional (*n* = 6)	CEBAS (*n* = 7)	*p* value
Gender (M/F)	2/4	2/5	0.7832
Age (years)	72 (68–83)	73 (58–78)	0.4472
BMI (kg/m^2^)	24.1 (16.7–28.0)	22.2 (18.0–32.2)	0.9913
History of diabetes	0	2 (29%)	0.0951
Operation time (min)	196 (164–256)	209 (182–233)	0.8303
Blood loss (ml)	10	10	
Type of surgery			
Iliocecal resection	2 (33%)	0	
Right/right hemi colectomy	4 (67%)	7 (100%)	
Length of small incision (cm)	3.5 (3–7)	4 (3–5)	0.6743
Tumor size (mm)	32 (23–75)	30 (10–74)	0.7375
Harvested lymph nodes (no.)	19 (13–33)	22 (12–31)	0.9577
TNM staging (no.)			0.3310
I	2 (33%)	3 (43%)	
IIa	4 (67%)	2 (29%)	
IIIa/IIIb	0	2 (29%)	
Days to first flatus	2 (1–3)	2 (1–4)	0.5548
Days to solid food	5 (3–7)	3 (3–8)	0.3340
Length of hospital stay	8 (6–10)	6 (6–20)	0.6379
Complications			
SSI	0	0	
Postoperative bleeding	0	0	
Ileus	1 (17%)	1 (14%)	0.9057
Leakage	0	0	

TNM stage is classified by UICC-8 staging Values are expressed as the median (range)

*BMI* body mass index, *SSI* surgical site infection

### Comparison of time to enterotomy closure

**Fig. 2:**
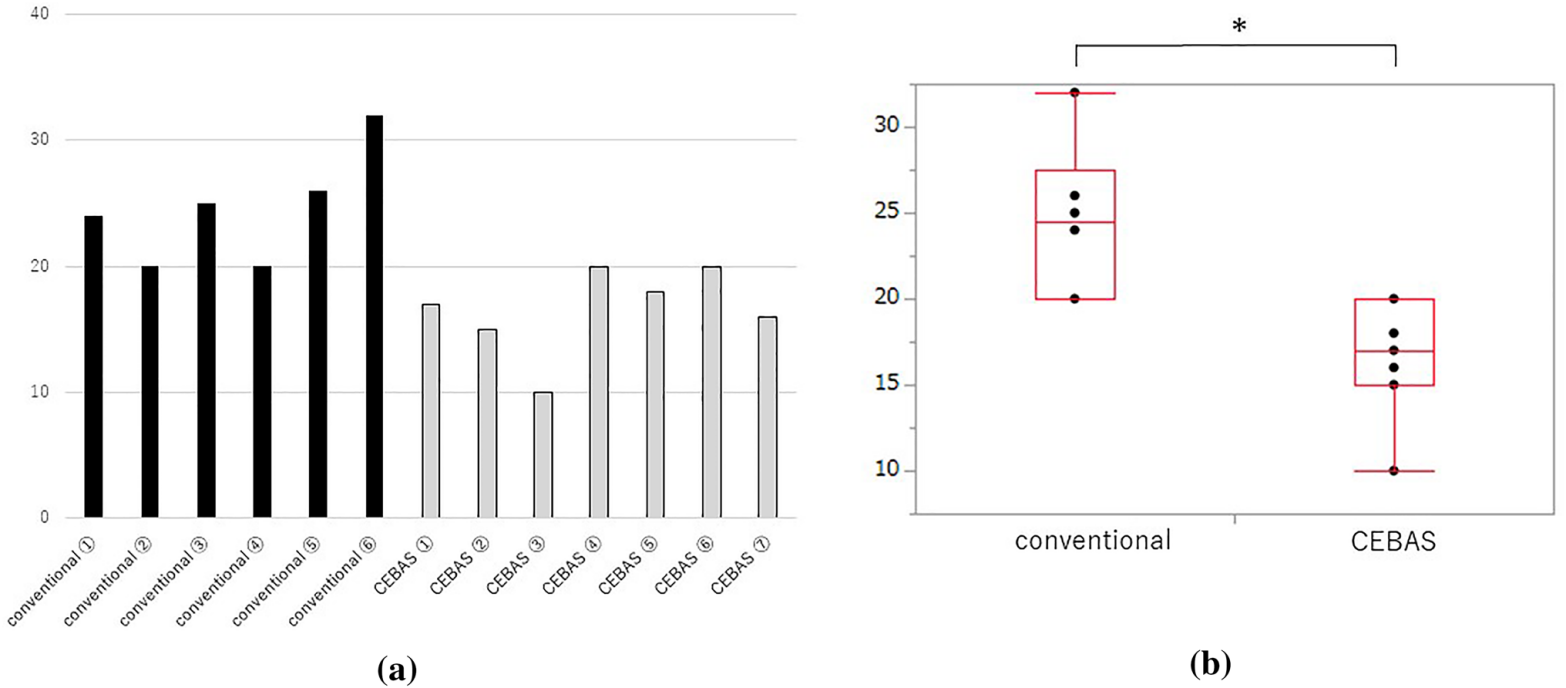
**a** Time to enterotomy closure in all patients. **b** Comparison of time to enterotomy closure between conventional and CEBAS groups. *p < 0.05

The time to enterotomy closure in each patient is shown in Fig. 2a. We found a significant difference in time to enterotomy closure (conventional vs. CEBAS: 24.5 ± 4.7 min vs. 16.5 ± 3.7 min, *p* = 0.0059) (Fig. 2b). The mean number of stitches was 10 in the first layer and 6 in the second layer for the conventional group. In the CEBAS group, the mean number of stitches was 10 in the first layer and 10 in the second layer.

## Discussion

Better short-term outcomes have been shown from IIA than from EIA in many retrospective reports [1, 2, 4–7], but no standardized technique has so far been described for remnant enterotomy closure after stapled side-to-side ileocolic anastomosis. Milone et al. [8] performed a multicenter case-controlled study using 1092 collected cases. That study recommended the adoption of double-layer enterotomy closure using a running barbed suture in the first layer because of the reduction in bleeding and leakages. We started TLRC with IIA in 2013 and enterotomy closure was performed with the Albert-Lembert method using a barbed suture and absorbable thread. The interrupted sutures in the second seromuscular layer using absorbable thread needed multiple intracorporeal ligations and were time-consuming and complicated, so we started the CEBAS method using a running barbed suture in the second seromuscular layer. The CEBAS method was not only feasible in terms of the short-term outcomes, but also shortened the anastomosis time significantly compared with the conventional method, despite the greater number of stitches in the second layer. The omission of ligation procedures might have influenced this result. No anastomotic bleeding or leakage was observed, and no patients developed surgical site infection. Although the observation period remains short, we have so far not encountered any cases of postoperative anastomotic stricture.

**Fig. 3:**
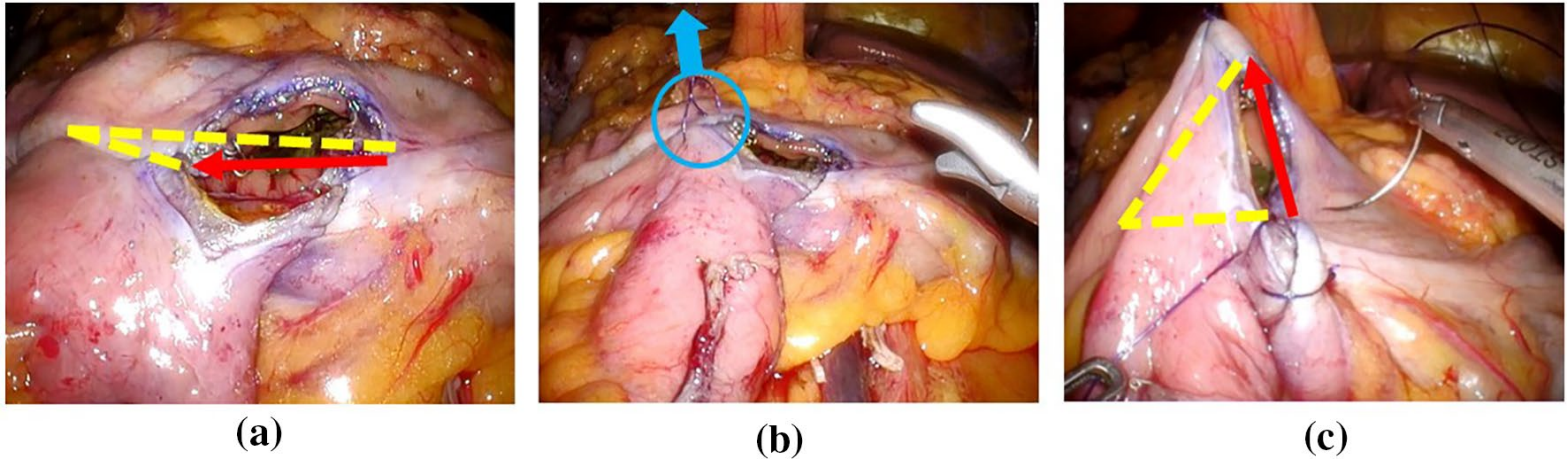
**a** After stapled side-to-side ileocolic anastomosis. **b** Pulling up in a cranial direction using the first barbed suture.**c** Anastomosis direction became a vertical axis. Yellow dotted line, staple line; red arrow, suture line.

Another benefit of the CEBAS method was that the direction of anastomosis became a vertical axis. After stapled side-to-side ileocolic anastomosis, we made a V-shaped anastomosis to avoid anastomotic stricture. Pulling up in a cranial direction using the first barbed suture made suturing easier and reduced the stress of sutures by aligning the suture axis (Fig. 3a–c).

A key limitation of this study was the retrospective, single-center design, but the CEBAS method proved to be a safe treatment modality and it may reduce the stress associated with intracorporeal enterotomy closure.

In conclusion, the CEBAS method in TLRC with IIA was found to be technically feasible. Further investigations are needed to assess the oncological outcomes associated with this technique.

## Electronic supplementary material

Below is the link to the electronic supplementary material.

Supplementary material 1 (MP4 83230 kb)
